# Dietary carbohydrate quality, fibre-rich food intake, and left ventricular structure and function: the CARDIA study

**DOI:** 10.1093/eurheartj/ehaf406

**Published:** 2025-07-08

**Authors:** So-Yun Yi, Lyn M Steffen, Weihua Guan, Daniel Duprez, Kamakshi Lakshminarayan, David R Jacobs

**Affiliations:** Division of Epidemiology and Community Health, University of Minnesota School of Public Health, 1300 South Second St, Suite 300, Minneapolis, MN 55454, USA; Division of Epidemiology and Community Health, University of Minnesota School of Public Health, 1300 South Second St, Suite 300, Minneapolis, MN 55454, USA; Division of Biostatistics, University of Minnesota School of Public Health, Minneapolis, MN, USA; Department of Medicine Cardiovascular Division, University of Minnesota, Minneapolis, MN, USA; Division of Epidemiology and Community Health, University of Minnesota School of Public Health, 1300 South Second St, Suite 300, Minneapolis, MN 55454, USA; Division of Epidemiology and Community Health, University of Minnesota School of Public Health, 1300 South Second St, Suite 300, Minneapolis, MN 55454, USA

**Keywords:** Carbohydrate to fibre ratio, Fibre, Cardiac phenotypes, Cardiac structure, Systolic function, Diastolic function

## Abstract

**Background and Aims:**

Identifying lifestyle risk factors related to abnormal cardiac phenotypes, including structure and function, will be essential to prevent or slow down the progression to heart failure. Little is known about intakes of macronutrients and food groups, particularly carbohydrate (CHO) quality and fibre-rich foods, relative to cardiac phenotypes. Therefore, the association between CHO quality and cardiac phenotypes was examined in the Coronary Artery Risk Development in Young Adults (CARDIA) study.

**Methods:**

Trained interviewers conducted the CARDIA Diet History to gather dietary intake at exam years 0, 7, and 20. Cardiac phenotype measures were collected at exam years 25 and 30 via echocardiography. Linear mixed effects regression models were used to evaluate the association of CHO quality and fibre-rich food score averaged across years 0, 7, and 20 with cardiac phenotype measures at years 25 and 30.

**Results:**

Among the 3171 CARDIA participants, quartiles of CHO quality (defined using CHO:fibre ratio) were favourably associated with left ventricular (LV) mass index (*P*_trend_ < .001) and global longitudinal strain (*P*_trend_ < .001) after adjusting for demographic and lifestyle factors. Similarly, quartiles of fibre-rich food score (created based on daily intakes of whole grains, fruit, vegetables, nuts, and legumes) were favourably associated with LV mass index (*P*_trend_ < .001), LV ejection fraction (*P*_trend_ = .008), global longitudinal strain (*P*_trend_ < .001), E/e′ ratio (*P*_trend_ = .02), and left atrial volume index (*P*_trend_ = .02). Cardiac phenotype effect sizes between 10% and 26% of their respective standard deviations.

**Conclusions:**

Higher quality of CHO and intake of fibre-rich foods were favourably associated with LV structure and function.

## Structured Graphical Abstract

**Graphical Abstract:**
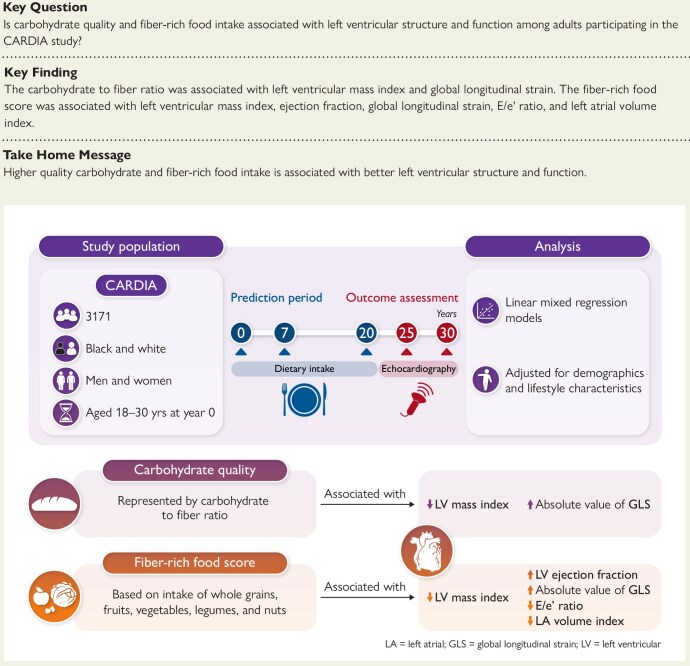
This figure summarizes the study design and main findings from the CARDIA study (n = 3,171), which included Black and White men and women. Higher carbohydrate quality was associated with lower left ventricular mass index and better global longitudinal strain. A higher fiber-rich food score was linked to lower left ventricular mass index, higher ejection fraction, better global longitudinal strain, lower E/e′ ratio, and smaller left atrial volume index.

**See the editorial comment for this article ‘Diet and heart failure: evidence is limited to make recommendations’, by A. Mente *et al*., https://doi.org/10.1093/eurheartj/ehaf521.**

Translational perspective Consuming a diet with high quality of carbohydrates (low carbohydrate:fibre ratio) was favourably associated with left ventricular structure and left ventricular systolic function, and fibre-rich food intake was favourably associated with left ventricular structure and left ventricular systolic and diastolic function. The fibre-rich food score was a better predictor of cardiac structure and function than the carbohydrate:fibre ratio, indicating that categorizing carbohydrates and fibre by food groups like grains, fruit, and vegetables offers a useful measure of carbohydrate quality, as both carbohydrate and fibre are predominantly present in plant-based foods.

## Introduction

Cardiovascular disease is the major cause of death globally.^1^ Heart failure (HF), in particular, affected about 64 million people worldwide in 2017.^2^ It should also be noted that many HF cases remain undiagnosed.^3^ In addition, it was impossible to distinguish between HF with reduced ejection fraction (HFrEF), HF with mid-range ejection fraction (HFmrEF), and HF with preserved ejection fraction (HFpEF) in the past. However, studies to date report that different mechanisms underlie HFrEF, HFmrEF, and HFpEF.^4,5^

Cardiac phenotypes, including cardiac structure and systolic and diastolic function, may serve as important predictors of progression to HF. Among US-based cohorts, the prevalence of abnormal cardiac phenotypes without diagnosed HF in middle-aged and older adults was approximately 25%–35%.^6–9^ Abnormal cardiac phenotypes are subclinical characteristics relevant for long-term assessment of HF risk as they can be detected before experiencing symptoms of HF. Identifying lifestyle risk factors related to abnormal cardiac phenotypes will be essential to prevent or slow down the progression to HF and ultimately reduce the burden of the ageing heart.

Dietary intake has been studied as one of the lifestyle risk factors for HF. The few studies that reported the associations between dietary patterns and cardiac phenotypes cannot be directly compared due to different exposure and outcome measures, but in general, healthier dietary patterns, such as plant-based diet patterns, were favourably associated with cardiac phenotype measures.^10^ Little is known about intakes of macronutrients and food groups, particularly carbohydrate (CHO) quality and fibre-rich foods, relative to cardiac phenotypes. Therefore, we aimed to examine the association between CHO quality and cardiac phenotypes among participants enrolled in the Coronary Artery Risk Development in Young Adults (CARDIA) study.

## Methods

Detailed information on the CARDIA study has been published elsewhere.^11^ Succinctly, CARDIA is a population-based longitudinal study that began in 1985–86 when it recruited Black and White race men and women aged 18–30 years to investigate the determinants and development of cardiovascular disease during young adulthood.

At baseline (year 0), 5115 participants free of definite cardiovascular disease were enrolled in the study. The field centres are located in Birmingham, AL (*n* = 1178); Chicago, IL (*n* = 1109); Minneapolis, MN (*n* = 1402); and Oakland, CA (*n* = 1426). Local Institutional Review Boards at the field centres approved the study protocols annually. At every clinic exam, all participants were asked to sign an informed consent.

### Eligibility and exclusion criteria

**Figure 1:**
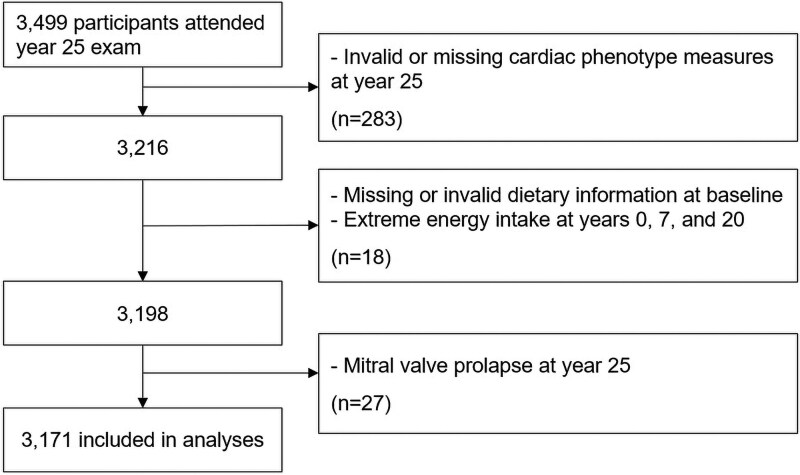
Flowchart of inclusion and exclusion criteria.

Participants who have dietary data at baseline and valid cardiac phenotype measures at year 25 were included in analyses. Those with mitral valve prolapse at year 25 were excluded from analyses. Participant data with extreme energy intake (<800 kcal/day or >8000 kcal/day for men and <600 kcal/day or >6000 kcal/day for women) and measurements taken during pregnancy were treated as missing for that exam year. A total of 3171 participants were included in this study. The inclusion and exclusion criteria flowchart is shown in *Figure 1*.

### Diet assessment

Trained interviewers assessed the usual dietary intake of the participants at years 0, 7, and 20 using the CARDIA Diet History. The Diet History is a tool asking about dietary intake over the past month.^12^ It included 100 closed-ended questions on food, beverages, and dietary supplements such as grains, fruits, vegetables, legumes, meat, fish and seafood, dairy products, candy, sugar-sweetened beverages, diet beverages, coffee, tea, and alcohol. If the participants answered affirmatively to the questions, the interviewers then obtained detailed information on food items from the participants including brand name, if available, how often (per day, week, or month) and how much they consumed the food item, how it was prepared, and whether they added anything to that food. To assist the participants in recalling the amount of food and beverage consumed, 3D food models were used. The validity and reliability of the CARDIA Diet History have been reported elsewhere.^13^

Data on total energy (kcal/day), food groups (serving/day), and nutrient values were obtained using the Nutrition Data System for Research. Nutrition Data System for Research is a software program developed at the University of Minnesota Nutrition Coordinating Center. Nutrition Data System for Research uses the US Department of Agriculture National Nutrient Database for Standard Reference as the primary source of food and nutrient composition. If food components and nutrient values are not available from the US Department of Agriculture and brand name food products, values from other reliable databases, scientific journal articles, and food manufacturers were utilized.^14^

### Carbohydrate quality

We utilized the CHO:fibre ratio as a measure of CHO quality, where a higher ratio indicates lower CHO quality. Various indices have been developed by researchers to assess the quality of CHOs in order to address their impact on health. Commonly reported indices include the glycaemic index, glycaemic load, CHO quality index, and different CHO ratios, such as the CHO:fibre ratio.^15^ Among these, the CHO:fibre ratio proved to be an effective and straightforward index for identifying processed CHO-rich foods with higher nutritional quality. A lower ratio indicates higher levels of protein and minerals and lower amounts of fat, added sugar, sodium, and calories.^16^ While the CHO:fibre ratio was initially designed to evaluate the quality of grain-based foods, we opted to use the overall CHO:fibre ratio from all food groups. This decision was made because fibre-rich foods, such as whole grains, fruit, vegetables, nuts, and legumes, are recognized for their health benefits. Additionally, the Dietary Guidelines for Americans recommend incorporating foods from these groups into one’s diet for a healthier eating pattern.^17^

In addition to the CHO:fibre ratio, we created a fibre-rich food score to examine the association between the consumption of fibre-rich foods and cardiac phenotypes. The food groups considered in this score include (i) whole grains, (ii) fruit, (iii) vegetables, (iv) nuts, and (v) legumes. To account for the remaining dietary intake, another food score was created using principal components analysis. This score focused on other foods and beverages, such as refined grains, candy, dairy, red and processed meat, poultry, eggs, fish and seafood, sugar-sweetened beverages, and diet beverages.

### Cardiac phenotypes

At years 25 and 30, Doppler echocardiography and 2D-guided M-mode echocardiography using an Artida cardiac ultrasound scanner (Toshiba Medical Systems, Japan) were performed by experienced sonographers using standardized protocols. Details on the quality control and reproducibility have been reported elsewhere.^18^

A standard off-line image analysis software system (Digisonics, Inc., Houston, Texas) was used to make measurements from digitized images. Left ventricular ejection fraction (LVEF) and left atrial volume were measured from the apical four-chamber view based on the American Society of Echocardiography guidelines. Left atrial volume index (LAVI) was calculated using left atrial volume and height (mL/m^2^). Left ventricular (LV) mass was derived from the Devereux formula using the American Society of Echocardiography guidelines.^19^ Left ventricular mass index (LVMI) was created based on LV mass and height (g/m^2.7^). Pulsed Doppler echocardiography recordings of transmitral flow were used to measure peak velocities of the early phase (E) and late phase (A) of the mitral inflow, and tissue Doppler imaging was used to measure early peak diastolic mitral annular velocity (e′) at the septal mitral annulus. Strain was calculated on the basis of speckle tracking as the peak systolic change in segment length relative to its end diastolic value. Global longitudinal strain (GLS) was defined as the average of segmental peaks measured from a six-segment model using the Advanced Cardiology Package 2D Wall Motion Tracking software system (version 3.0, Toshiba Medical Systems, Tochigi, Japan). Global longitudinal strain reflects the shortening of the LV in the longitudinal planes, and it is typically expressed as a negative value. Lower negative values (smaller absolute value) suggest reduced shortening, indicating a greater impairment in myocardial contractility. The LV relative wall thickness was calculated by dividing the sum of ventricular septum and posterior wall thicknesses by the LV internal diameter during diastole.

In this study, we used (i) LVMI, (ii) LVEF and GLS, and (iii) E/e′ ratio and LAVI to represent LV (i) structure, (ii) systolic function, and (iii) diastolic function, respectively. The rationale for selecting the aforementioned measurements is reported in Supplementary data online, *Table S1*.

As an additional analysis, LV remodelling at year 30 was examined. Normal was defined as LVMI ≤ 51 g/m^2.7^ and relative wall thickness < .42. Concentric remodelling was defined as LVMI ≤ 51 g/m^2.7^ and relative wall thickness ≥ .42. Left ventricular hypertrophy with eccentric remodelling was defined as LVMI > 51 g/m^2.7^ and relative wall thickness < .42, and LV hypertrophy with concentric remodelling was defined as LVMI > 51 g/m^2.7^ and relative wall thickness ≥ .42. In short, LV hypertrophy refers to an increased LV mass, while ‘concentric’ indicates thickened LV walls and ‘eccentric’ indicates thin LV walls.

### Demographic, lifestyle, and clinical factors

Demographic characteristics (age, sex, race, and education), lifestyle factors (physical activity, smoking, and alcoholic beverage drinking habits), medical history, and medication use (for hypertension, lipid lowering, or diabetes) were obtained using standardized questionnaires. To quantify physical activity, a score was calculated based on the total time spent on activities performed during work and leisure time, weighted by estimated energy expenditure per minute.^20^ Weight was measured using a beam balance scale, and height was measured using a stadiometer. Body mass index (BMI) was calculated based on weight and height (kg/m^2^). Blood pressure during sitting rest was measured three times, and the last two measures were averaged for each of systolic blood pressure and diastolic blood pressure. A random zero sphygmomanometer was used for years 0 through 15, and then, an Omron oscillometer, calibrated to the random zero device, was used from year 20.

### Statistical methods

SAS software was used for all data analyses in this study. To determine statistical significance, we used an alpha level of .05 for LVMI and Bonferroni adjusted alpha level of .025 for systolic and diastolic function measures to account for multiple comparisons. To evaluate the associations between CHO quality (averaged across years 0, 7, and 20) and cardiac phenotypes repeated at years 25 and 30, linear mixed effects models were used adjusting for a set of potential confounders. We included random intercepts for individuals to account for repeated measures over time, treated CHO quality as a fixed effect, and used compound symmetry as the covariance structure. Multinomial logistic regression was used to assess the association between quartiles of CHO quality (averaged at years 0, 7, and 20) and LV remodelling at year 30.

The intakes of total CHO (g/day) was divided by total dietary fibre (g/day) to create CHO:fibre ratio. Dietary data, including the CHO:fibre ratio, at years 0, 7, and 20 were averaged to represent the overall dietary intake during follow-up. Despite the presence of missing data at years 7 and/or 20 for some participants, we chose to use the averaged dietary data, incorporating whatever information was available. The averaged CHO:fibre ratio was inversely ranked to create quartiles of CHO quality (higher quartile representing higher CHO quality). The fibre-rich food score was created based on the servings of each fibre-rich food group (whole grains, fruits, vegetables, nuts, and legumes),^21^ ranked in quintiles. Scores from each food group were then added together to generate the fibre-rich food score (score range: 0–20). Then, the fibre-rich food score was ranked in quartiles to be included in the analysis models. The cardiac phenotype measures, except for GLS, underwent log transformation for data normalization. Back-transformed means and standard errors were then presented to illustrate the strength and direction of the associations.

We adjusted Model 1 for time, age, sex, race, field centre, education, and energy intake and Model 2 for Model 1 plus physical activity, alcohol drinking, smoking, supplement use, and medication use. The same covariates were done for fibre-rich food score analyses, except that Model 2 was further adjusted for a principal components analysis-derived food score of intakes of remaining foods and beverages. Model 3 further adjusted Model 2 for BMI, diabetes, and systolic blood pressure, factors in the causal pathway of developing adverse cardiac phenotypes. We examined effect modification by including cross-product terms in the models and verified that there was no effect modification by time, race, sex, and obesity (*P* > .10).

## Results

Study participants were 25.1 years old on average at baseline, 57.0% female, and 59.9% White race. The mean CHO:fibre ratio and the fibre-rich food score were 17.3 and 9.8, respectively. The correlation coefficients between repeated measurements of CHO:fibre ratio and of cardiac phenotypes are reported in Supplementary data online, *Tables S2* and *S3*, respectively. Participants who had the highest CHO quality were more likely to be older, female, White race, educated, physically active, current drinker, non-current smoker, and supplement user and have lower BMI compared with those who had the lowest CHO quality (*Table 1*). Clinical characteristics at year 30 and participant characteristics by inclusion and exclusion status are reported in Supplementary data online, *Tables S4* and *S5*. As shown in *Table 2*, participants who had the highest CHO quality had higher intakes of fibre, protein, whole grains, fruit, vegetables, nuts, legumes, poultry, fish/seafood, and diet beverages and lower intakes of energy, added sugar, saturated fat, refined grains without desserts, sugar-sweetened beverages, candy, dairy products, and red and processed meat.

**Table 1:** Unadjusted means (SD) of participant characteristics at baseline (year 0) and cardiac phenotype measures at year 25 stratified by quartiles of CHO quality (*n* = 3171)

	CHO quality			
	Q1 (low quality) (*n* = 793)	Q2 (*n* = 793)	Q3 (*n* = 793)	Q4 (high quality) (*n* = 792)
CHO:fibre ratio^a^, median (range)	23.6 (20.1+)	17.8 (16.0–20.1)	14.5 (13.0–15.9)	11.3 (5.9–12.9)
Age, y	23.7 (3.73)	24.8 (3.64)	25.5 (3.47)	26.3 (3.09)
Female, *n* (%)	394 (49.7)	400 (50.4)	448 (56.5)	565 (71.3)
White race, *n* (%)	251 (31.7)	340 (42.9)	474 (59.8)	613 (77.4)
Education, y	13.0 (1.92)	13.7 (2.09)	14.5 (2.3)	15.2 (2.13)
Physical activity	380 (296.5)	404 (302.6)	432 (300.8)	469 (287.2)
Current drinker, *n* (%)	659 (83.1)	682 (86.0)	707 (89.2)	705 (89.0)
Current smoker, *n* (%)	276 (34.8)	216 (27.2)	174 (21.9)	146 (18.4)
BMI, kg/m^2^	24.5 (5.25)	24.4 (4.73)	24.5 (4.55)	23.6 (4.30)
Diabetes, *n* (%)	9 (1.1)	1 (.1)	7 (.9)	5 (.6)
SBP, mmHg	110.7 (10.6)	110.4 (10.7)	110.2 (10.8)	107.6 (10.3)
Supplement use, *n* (%)	177 (22.3)	253 (31.9)	344 (43.4)	426 (53.8)
Medication use^b^, *n* (%)	31 (3.9)	14 (1.8)	35 (4.4)	17 (2.2)
Cardiac phenotypes				
LVMI, g/m^2.7^	35.2 (9.99)	34.2 (9.35)	33.3 (8.83)	31.5 (8.03)
LVEF, %	69.5 (8.33)	69.2 (8.45)	69.2 (8.01)	69.9 (7.55)
GLS^c^, %	−14.64 (2.44)	−14.85 (2.44)	−15.16 (2.39)	−15.65 (2.32)
E/e′ ratio	9.39 (2.98)	9.21 (2.84)	8.79 (2.76)	8.44 (2.45)
LAVI, mL/m^2^	17.4 (5.50)	17.1 (5.29)	17.2 (5.08)	16.6 (5.09)

BMI, body mass index; LVMI, left ventricular mass index; LVEF, left ventricular ejection fraction; GLS, global longitudinal strain; LAVI, left atrial volume index; SBP, systolic blood pressure.

^a^The CHO:fibre ratio was created by dividing the intakes of total CHO (g/day) by total dietary fibre (g/day). The average across available measures at year 0, 7, and 20 is presented.

^b^Medication use for hypertension, lipid lowering, and or diabetes.

^c^Lower negative values (smaller absolute value) suggest reduced shortening, indicating a greater impairment in myocardial contractility.

**Table 2:** Adjusted means (SE) of averaged (years 0, 7, and 20) dietary intake^a^ stratified by quartiles of CHO quality (*n* = 3171)

	CHO quality			
	Q1 (low quality) (*n* = 793)	Q2 (*n* = 793)	Q3 (*n* = 793)	Q4 (high quality) (*n* = 792)
CHO:fibre ratio^b^, median (range)	23.6 (20.1+)	17.8 (16.0–20.1)	14.5 (13.0–15.9)	11.3 (5.9–12.9)
Nutrients				
Energy, kcal	2837 (33.8)	2816 (32.3)	2638 (32.5)	2601 (35.6)
Total CHO, g	319.5 (1.62)	305.0 (1.55)	305.5 (1.56)	313.9 (1.70)
Fibre, g	11.4 (.23)	14.0 (.22)	16.52 (.22)	20.8 (.24)
Added sugar, g	110.3 (1.24)	81.3 (1.19)	70.0 (1.19)	60.6 (1.30)
Protein, g	93.1 (.77)	99.5 (.73)	102.5 (.74)	105.0 (.81)
Total fat, g	110.1 (.93)	112.6 (.89)	113.4 (.90)	111.7 (.98)
Saturated fat, g	40.5 (.38)	40.1 (.36)	39.8 (.37)	37.1 (.40)
Food groups, (servings/day)				
Whole grains	.07 (.01)	.12 (.01)	.17 (.01)	.32 (.01)
Fruit	2.40 (.08)	2.78 (.07)	3.24 (.07)	3.96 (.08)
Vegetables	2.86 (.06)	3.59 (.06)	4.15 (.06)	5.66 (.07)
Nuts	.44 (.04)	.69 (.04)	.87 (.04)	1.27 (.04)
Legumes	.13 (.02)	.23 (.02)	.31 (.02)	.56 (.02)
Refined grain desserts	.14 (.01)	.18 (.01)	.16 (.01)	.18 (.01)
Refined grains without desserts	4.50 (.06)	4.14 (.06)	3.81 (.06)	3.35 (.06)
SSBs	3.79 (.07)	2.73 (.07)	2.47 (.07)	2.30 (.08)
Candy	.39 (.02)	.32 (.01)	.29 (.01)	.23 (.02)
Dairy products	2.81 (.06)	2.75 (.06)	2.81 (.06)	2.38 (.06)
Red/proc meat	3.61 (.07)	3.73 (.06)	3.50 (.06)	3.06 (.07)
Poultry	1.19 (.04)	1.36 (.04)	1.35 (.04)	1.52 (.04)
Eggs	.59 (.02)	.58 (.02)	.60 (.02)	.63 (.02)
Fish/seafood	.78 (.04)	1.01 (.03)	1.09 (.03)	1.26 (.04)
Diet beverages	.43 (.04)	.65 (.04)	.72 (.04)	.63 (.04)

CHO, carbohydrate; SSBs, sugar-sweetened beverages.

^a^Adjusted for age, sex, race, education, field centre, and energy intake.

^b^The CHO:fibre ratio was created by dividing the intakes of total CHO (g/day) by total dietary fibre (g/day).

Higher CHO quality was favourably associated with LVMI (*P*_trend_ < .001) and GLS (*P*_trend_ < .001), while LVEF, E/e′ ratio, and LVAI were not associated with CHO quality (*Table 3*). The differences of LVMI and GLS between Q4 and Q1 were −2.0 g/m^2.7^ [22% of standard deviation (SD)] and −.62% points (26% of SD), respectively. The associations between CHO quality and cardiac phenotype measures remained the same after further adjustment for BMI, diabetes, and systolic blood pressure (data not shown). The fibre-rich food score was favourably associated with LVMI (*P*_trend_ < .001), LVEF (*P*_trend_ = .008), GLS (*P*_trend_ < .001), E/e′ ratio (*P*_trend_ = .02), and LAVI (*P*_trend_ = .02) (*Table 4*). The differences of the aforementioned measures between Q4 and Q1 were −2.3 g/m^2.7^ (25% of SD), 1.1% points (14% of SD), −.49% points (20% of SD), −.27 (10% of SD), and −.7 mL/m^2^ (13% of SD). When further adjusted for BMI, diabetes, and systolic blood pressure, LAVI was no longer associated with the fibre-rich food score (*P*_trend_ = .06), while the associations between the fibre-rich food score and other cardiac phenotype measures remained the same (data not shown). Unadjusted models are shown in Supplementary data online, *Table S6*.

**Table 3:** Adjusted means (SE) of cardiac phenotype measures^a,b^ stratified by quartiles of averaged (years 0, 7, and 20) CHO quality (*n* = 3171)

	CHO quality				*P*_trend_
	Q1 (low quality) (*n* = 793)	Q2 (*n* = 793)	Q3 (*n* = 793)	Q4 (high quality) (*n* = 792)	
CHO:fibre ratio^c^, median (range)	23.6 (20.1+)	17.8 (16.0–20.1)	14.5 (13.0–15.9)	11.3 (5.9–12.9)	
Structure					
LVMI, g/m^2.7^	37.6 (1.02)	37.2 (1.02)	37.0 (1.02)	35.6 (1.02)	<.001
Systolic function					
LVEF, %	65.6 (1.01)	65.6 (1.01)	65.6 (1.01)	65.7 (1.01)	.75
GLS^d^, %	−14.52 (.18)	−14.87 (.18)	−14.90 (.17)	−15.14 (.17)	<.001
Diastolic function					
E/e′ ratio	9.03 (1.02)	8.86 (1.02)	8.83 (1.02)	8.75 (1.02)	.07
LAVI, mL/m^2^	17.0 (1.02)	17.1 (1.01)	17.2 (1.02)	16.8 (1.01)	.42

CHO, carbohydrates; LVMI, left ventricular mass index; LVEF, left ventricular ejection fraction; GLS, global longitudinal strain; LAVI, left atrial volume index.

^a^Adjusted for time, age, sex, race, education, field centre, energy intake, physical activity, drinking, smoking, supplement use, and medication use. Adjusted means tabulated from the mixed model.

^b^SD of year 25 cardiac structure and function measures: LVMI = 9.17; LVEF = 8.09; GLS = 2.43; E/e′ ratio = 2.79; LAVI = 5.25.

^c^The CHO:fibre ratio was created by dividing the intakes of total CHO (g/day) by total dietary fibre (g/day).

^d^Lower negative values (smaller absolute value) suggest reduced shortening, indicating a greater impairment in myocardial contractility.

**Table 4:** Adjusted means (SE) of cardiac phenotype measures^a,b^ stratified by quartiles of averaged (years 0, 7, and 20) fibre-rich food score (*n* = 3171)

	Fibre-rich food score				*P*_trend_
	Q1 (low fibre) (*n* = 793)	Q2 (*n* = 740)	Q3 (*n* = 908)	Q4 (high fibre) (*n* = 730)	
Fibre-rich rood score^c^, mean (range)	4.0 (0–6)	8.0 (7–9)	11.4 (10–13)	16.1 (14–20)	
Structure					
LVMI, g/m^2.7^	38.3 (1.02)	37.3 (1.02)	36.6 (1.02)	36.0 (1.02)	<.001
Systolic function					
LVEF, %	65.2 (1.01)	65.6 (1.01)	65.9 (1.01)	66.3 (1.01)	.008
GLS^d^, %	−14.60 (.15)	−14.76 (.15)	−14.91 (.14)	−15.09 (.15)	<.001
Diastolic function					
E/e′ ratio	9.07 (1.02)	8.91 (1.02)	8.75 (1.02)	8.80 (1.02)	.02
LAVI, mL/m^2^	17.4 (1.02)	17.2 (1.02)	16.8 (1.02)	16.7 (1.02)	.02

LVMI, left ventricular mass index; LVEF, left ventricular ejection fraction; GLS, global longitudinal strain; LAVI, left atrial volume index.

^a^Adjusted for time, age, sex, race, education, field centre, energy intake, physical activity, principal components analysis-derived food score, drinking, smoking, supplement use, and medication use. Adjusted means tabulated from the mixed model.

^b^SD of year 25 cardiac structure and function measures: LVMI = 9.17; LVEF = 8.09; GLS = 2.43; E/e′ ratio = 2.79; LAVI = 5.25.

^c^The fibre-rich food score was created based on daily intakes of (i) whole grains, (ii) fruit, (iii) vegetables, (iv) nuts, and (v) legumes.

^d^Lower negative values (smaller absolute value) suggest reduced shortening, indicating a greater impairment in myocardial contractility.

The regression coefficients (*β*) per 1 SD increment of CHO:fibre ratio and fibre-rich food score for describing cardiac structure and function measures were reported in Supplementary data online, *Table S7*. Briefly, CHO:fibre ratio was positively associated with GLS (*β* per 1 SD = .07% points, *P* = .02) and E/e′ ratio (*β* per SD = .14, *P* = .007), while the fibre-rich food score was negatively associated with LVMI (*β* per SD = −.91 g/m^2.7^  *P* < .001), GLS (*β* per SD = −.18% points, *P* < .001), E/e′ ratio (*β* per SD = −.14, *P* = .005), and LAVI (*β* per SD = −.23 mL/m^2^, *P* < .001) and positively associated with LVEF (*β* per SD = .36% points, *P* = .02). In addition, higher CHO quality and higher fibre-rich food score were associated with less cardiac remodelling (see Supplementary data online, *Table S8*).

## Discussion

Higher quality of CHO (represented by lower CHO:fibre ratio) was favourably associated with LV structure and LV systolic function, and a higher fibre-rich food score was favourably associated with LV structure and LV systolic and diastolic function after adjusting for demographic and lifestyle factors. Also, with increasing LV mass, cardiac remodelling of various types was observed, and they were less common with a better diet.

The favourable associations of higher CHO quality and diet high in fibre-rich foods with cardiac phenotypes may be explained by the effects of a myriad of nutrients that act synergistically within plant-based diets to reduce oxidative and inflammatory stress.^22^ In CARDIA, metabolite signatures of a diet pattern low in fibre-rich foods were associated with long-term cardiometabolic and cardiovascular disease.^23^ Additionally, the CHO:fibre ratio has been associated with diabetes,^24^ coronary heart disease,^25^ and systolic blood pressure,^26^ which are potentially in the causal pathway between CHO quality and cardiac phenotypes.^27^

Though studies on CHO quality and cardiac phenotypes are scarce, there are studies that reported individual food groups, nutrients, and dietary patterns associated with health outcomes related to cardiac phenotypes and HF. Higher intakes of whole grains and fruit were associated with a lower risk of HF, while intakes of vegetables and nuts were not associated with risk of HF in a meta-analysis of observational studies.^28^ In a meta-analysis of 24 randomized placebo-controlled trials, fibre supplementation lowered systolic and diastolic blood pressure by 1.13 and 1.26 mmHg, respectively.^29^ In addition, dietary fibre has been associated with inflammation measures that are associated with heart function and cardiovascular disease including HF.^30^ Dietary patterns high in fibre including the Mediterranean diet and the Dietary Approaches to Stop Hypertension (DASH) diet have been associated with better clinical outcomes such as mortality, blood pressure, and cardiac systolic and diastolic function in patients with HF.^31^ Further studies on the associations of CHO quality and fibre-rich food intake with subclinical markers such as heart rate recovery,^32^ Pathobiologic Determinants of Atherosclerosis in Youth (PDAY) risk score,^33^ and coronary artery calcium, to name a few, are needed to better understand how dietary factors are related to cardiovascular health and potential for early detection of HF and other cardiovascular diseases.

There are several limitations of this study. First, self-reported dietary intake is subject to measurement error. However, the CARDIA Diet History captures usual dietary intake better than most dietary assessment tools, such as a food frequency questionnaire, as the participants are asked to report individual foods and beverages consumed along with detailed information such as brand name, preparation and cooking methods, and additions to foods and beverages. Second, even though potential confounders were included in the statistical models, we cannot rule out the possibility of residual confounding by factors not collected in the CARDIA study. Although we cannot establish the causal relationship due to the nature of the observational study design, we assumed that the participants did not modify their diets due to cardiac phenotypes as most participants’ cardiac phenotype measures fall in the normal range and the participants are relatively young to have symptomatic HF. Third, this study examines two exposures and five outcome measures which can raise a multiple comparisons issue. However, given the high correlation between the CHO:fibre ratio and the fibre-rich food score (correlation coefficient = −.46), they may be considered as a replicated test rather than independent tests. The Bonferroni correction *P*-value limit for ten tests is .005; our strongly significant findings have conventional *P* < .001, so would be significant even with this conservative decision rule. Also, the outcome measures were carefully selected during the study design phase, and other echocardiography measures were not tested in this study. Fourth, there has been loss-to-follow-up during the long years of the CARDIA study. There were small differences in that those excluded were more likely to be male, Black race, less educated, and smokers (see Supplementary data online, *Table S5*). Last, there is an issue of generalizability as only Black and White race in four sites in the USA were enrolled in the CARDIA study. However, our study has several strengths. First, the coverage of our data is improved by the repeated measures of diet and cardiac phenotype measures; thus, noise is reduced assuming that there is no real change. Second, as the CARDIA study included biracial men and women, we were able to examine whether the associations of dietary intake and cardiac phenotypes differ by sex or race. Third, the mixed effects models we used are conditioned to handle missing data through likelihood-based methods.

## Conclusions

The fibre-rich food score was more predictive than the CHO:fibre ratio regarding the association with cardiac phenotypes. Categorizing CHO and dietary fibre based on food groups like grains, fruits, and vegetables provides a valuable indicator for assessing CHO quality, as both CHO and fibre are predominantly present in plant-based foods.^15^ Therefore, our study findings support the dietary guidance to improve cardiovascular health and cardiovascular disease prevention by the American Heart Association^34^ and the European Society of Cardiology^35^ that recommend plant-based diet patterns and intakes of whole grains, fruits, vegetables, nuts, and legumes (pulses).

## Supplementary Material

ehaf406_Supplementary_Data

## Declarations

### Data Availability

CARDIA complies with data sharing requirements of the National Institutes of Health by providing limited-access data sets from various CARDIA examinations to the National Heart, Lung and Blood Institute BioLINCC. Data are available through BioLINCC.

### Pre-registered Clinical Trial Number

None supplied.
