# Supplementary material for: Dietary carbohydrate quality, fibre-rich food intake, and left ventricular structure and function: the CARDIA study
Source: Eur Heart J. 2025 Jul 8;46(41):4329–37. doi: 10.1093/eurheartj/ehaf406 (PMC12579980; doi:10.1093/eurheartj/ehaf406)
Supplement: ehaf406_Supplementary_Data [file ehaf406_supplementary_data.pdf]

Supplemental Table 1. Rationale for echocardiography measurement selection.

	Echocardiography measure	Rationale
**Structure**	LVMI, $g/m^{2.7}$	• Represents the cumulative effect of blood pressure on the heart.<br>• Parameter used to determine LV hypertrophy.
**Systolic function**	LVEF, %	• Most used systolic function parameter.
	GLS, %	• LS is sensitive to LV dysfunction[1] and is a superior predictor of major adverse cardiac events than LVEF.[2]
**Diastolic function**	E/e' ratio	• Index of left ventricular filling pressures.<br>• Regarded as the most important diastolic function parameter.[3]
	LAVI, mL/m^2	• Reflects the cumulative effects on the left atrium of increased LV filling pressures over time.<br>• Provides diagnostic and prognostic details regarding LV diastolic dysfunction and chronicity of disease.[4]<br>• An independent predictor of mortality, heart failure, atrial fibrillation, and ischemic stroke.[4]

LVMI = left ventricular mass index; LVEF = left ventricular ejection fraction; GLS = global longitudinal strain; LAVI = left atrial volume index.

[1] Potter E, Huynh Q, Haji K, et al. Use of Clinical and Echocardiographic Evaluation to Assess the Risk of Heart Failure. JACC Heart Fail. doi:10.1016/j.jchf.2023.06.014
[2] Kalam K, Otahal P, Marwick TH. Prognostic implications of global LV dysfunction: a systematic review and meta-analysis of global longitudinal strain and ejection fraction. Heart. 2014;100(21):1673-80. doi:10.1136/heartjnl-2014-305538
[3] Hummel YM, Klip IJT, De Jong RM, Pieper PG, Van Veldhuisen DJ, Voors AA. Diastolic function measurements and diagnostic consequences: a comparison of pulsed wave- and color-coded tissue Doppler imaging. Clinical Research in Cardiology. 2010;99(7):453-458. doi:10.1007/s00392-010-0141-y
[4] Nagueh SF, Smiseth OA, Appleton CP, et al. Recommendations for the Evaluation of Left Ventricular Diastolic Function by Echocardiography: An Update from the American Society of Echocardiography and the European Association of Cardiovascular Imaging. Journal of the American Society of Echocardiography. 2016;29(4):277-314. doi:10.1016/j.echo.2016.01.011

Supplemental Table 2. Spearman correlation coefficients between year 0, year 7, and year 20 carbohydrate:fiber ratio

	Year 0	Year 7	Year 20
**Year 0**		0.44	0.44
**Year 7**			0.46
**Year 20**			

[b] The carbohydrate:fiber ratio was created by dividing the intakes of total carbohydrate (g/day) by total dietary fiber (g/day).

Supplemental Table 3. Pearson correlation coefficients between year 25 and year 30 cardiac phenotype measures

	Correlation coefficient	p
**LVMI**	0.61	<0.001
**LVEF**	0.34	<0.001
**GLS**	0.33	<0.001
**E/e' ratio**	0.33	<0.001
**LAVI**	0.48	<0.001

LVMI = left ventricular mass index; LVEF = left ventricular ejection fraction; GLS = global longitudinal strain; LAVI = left atrial volume index.

Supplemental Table 4. Unadjusted clinical characteristics at year 30 stratified by quartiles of CHO quality (n=3,171)

	**CHO Quality**			
	**Q1 (Low quality) (n=793)**	**Q2 (n=793)**	**Q3 (n=793)**	**Q4 (High quality) (n=792)**
CHO:fiber ratio[a], median (range)	23.6 (20.1+)	17.8 (16.0 – 20.1)	14.5 (13.0 – 15.9)	11.3 (5.9 – 12.9)
BMI, kg/m^2	30.8 (6.8)	30.6 (7.3)	30.9 (7.2)	31.1 (7.2)
Diabetes, n (%)	99 (12.5)	90 (11.3)	80 (10.1)	92 (11.6)
SBP, mmHg	121.1 (16.8)	120.8 (17.4)	121.1 (17.0)	122.0 (17.7)

BMI = body mass index; CHO = carbohydrates; SBP = systolic blood pressure.

Supplemental Table 5. Unadjusted means (SD) of participant characteristics at baseline (year 0) by inclusion and exclusion status (n=5,115).

	**Included (n=3,171)**	**Excluded (n=1,944)**
Age, y	25.1 (3.61)	24.5 (3.71)
Female, n (%)	1807 (57.0)	980 (50.4)
White race, n (%)	1678 (52.9)	800 (41.2)
Education, y	14.1 (2.27)	13.3 (2.13)
Physical activity	421 (299)	418 (304)
Current drinker, n (%)	2753 (86.8)	1652 (85.0)
Current smoker, n (%)	812 (25.6)	734 (37.8)
BMI, kg/m^2	24.3 (4.73)	24.9 (5.50)
Diabetes, n (%)	22 (0.7)	21 (1.1)
SBP, mmHg	109.7 (10.7)	111.6 (11.3)
Supplement use, n (%)	1200 (37.8)	630 (32.4)
Medication use[a], n (%)	97 (3.1)	81 (4.2)

BMI = body mass index; SBP = systolic blood pressure.

[a] Medication use for hypertension, lipid-lowering, and or diabetes

Table 6. Unadjusted means (SE) of cardiac structure and function measures stratified by quartiles of averaged (years 0, 7, and 20) fiber-rich food score (n=3,171)

	**Fiber-rich food score**				
	**Q1 (Low fiber) (n=793)**	**Q2 (n=740)**	**Q3 (n=908)**	**Q4 (High fiber) (n=730)**	p_{trend}
Fiber-rich rood score[a], mean (range)	4.0 (0-6)	8.0 (7-9)	11.4 (10-13)	16.1 (14-20)	
**Structure**					
LVMI, $g/m^{2.7}$	35.9 (1.01)	34.9 (1.01)	34.2 (1.01)	33.2 (1.01)	<0.001
**Systolic function**					
LVEF, %	66.3 (1.00)	66.6 (1.00)	66.6 (1.00)	66.8 (1.00)	0.21
GLS[b], %	-14.8 (0.08)	-15.0 (0.08)	-15.1 (0.08)	-15.4 (0.08)	<0.001
**Diastolic function**					
E/e' ratio	9.06 (1.01)	8.80 (1.01)	8.54 (1.01)	8.39 (1.01)	<0.001
LAVI, mL/m^2	16.9 (1.01)	16.9 (1.01)	16.4 (1.01)	16.3 (1.01)	0.003

LVMI = left ventricular mass index; LVEF = left ventricular ejection fraction; GLS = global longitudinal strain; LAVI = left atrial volume index.

[a] The fiber-rich food score was created based on daily intakes of 1) whole grains, 2) fruit, 3) vegetables, 4) nuts, and 5) legumes.

[b] Lower negative values (smaller absolute value) suggest reduced shortening, indicating a greater impairment in myocardial contractility.

Supplemental Table 7. Regression coefficients (β) per 1 SD increment of CHO:fiber ratio and fiber-rich food score for describing cardiac phenotype measures[a] (n=3,171)

	**CHO:fiber ratio[b]**		**Fiber-rich food score[d]**	
	**β (SE)[c]**	**p**	**β (SE)[e]**	**p**
**Structure**				
LVMI, $g/m^{2.7}$	0.28 (0.007)	0.14	-0.91 (0.23)	<0.001
**Systolic function**				
LVEF, %	0.07 (0.14)	0.70	0.36 (0.14)	0.02
GLS[e], %	0.07 (0.07)	0.02	-0.18 (0.05)	<0.001
**Diastolic function**				
E/e' ratio	0.14 (0.07)	0.007	-0.14 (0.05)	0.005
LAVI, mL/m^2	0.014 (0.07)	0.90	-0.23 (0.09)	0.02

CHO = carbohydrates; LVMI = left ventricular mass index; LVEF = left ventricular ejection fraction; GLS = global longitudinal strain; LAVI = left atrial volume index.

[a] Cardiac phenotype measures were not log-transformed.

[b] The CHO:fiber ratio was created by dividing the intakes of total CHO (g/day) by total dietary fiber (g/day).

[c] Adjusted for time, age, sex, race, education, field center, energy intake, physical activity, drinking, smoking, supplement use, and medication use.

[d] The fiber-rich food score was created based on daily intakes of 1) whole grains, 2) fruit, 3) vegetables, 4) nuts, and 5) legumes.

[e] Adjusted for time, age, sex, race, education, field center, energy intake, physical activity, principal components analysis -derived food score, drinking, smoking, supplement use, and medication use.

[f] Lower negative values (smaller absolute value) suggest reduced shortening, indicating a greater impairment in myocardial contractility.

Supplemental Table 8. Odds ratios and 95% confidence intervals of cardiac remodeling[a] (year 30) stratified by quartiles of averaged (years 0, 7, and 20) CHO quality.

	CHO Quality			
	**Q1 (Low quality)**	**Q2**	**Q3**	**Q4 (High quality)**
**Concentric Remodeling[b]**				
Odds Ratio	Ref	0.85	0.79	0.62
95% CI	-	0.63 – 1.15	0.58 – 1.09	0.43 – 0.88
**LVH with Eccentric Remodeling[c]**				
Odds Ratio	Ref	0.72	0.67	0.50
95% CI	-	0.44 – 1.16	0.40 – 1.23	0.72 – 0.94
**LVH with Concentric Remodeling[d]**				
Odds Ratio	Ref	0.77	0.68	0.55
95% CI	-	0.48 – 1.23	0.41 – 1.13	0.30 – 1.03
	**Fiber-rich food score**			
	**Q1 (Low fiber)**	**Q2**	**Q3**	**Q4 (High fiber)**
**Concentric Remodeling[b]**				
Odds Ratio	Ref	0.99	0.82	0.69
95% CI	-	0.73 – 1.37	0.60 – 1.13	0.47 – 1.01
**LVH with Eccentric Remodeling[c]**				
Odds Ratio	Ref	0.73	0.66	0.40
95% CI	-	0.44 – 1.21	0.39 – 1.10	0.20 – 0.77
**LVH with Concentric Remodeling[d]**				
Odds Ratio	Ref	1.06	0.67	0.57
95% CI	-	0.48 – 1.23	0.39 – 1.09	0.29 – 1.10

CHO = carbohydrates; LVH = Left ventricle hypertrophy; LVMI = Left ventricular mass index.

[a] Adjusted for age, sex, race, field center, education, energy intake, physical activity, drinking, smoking, supplement use, and medication use. Reference group: normal, LVMI ≤ 51 $g/m^{2.7}$ and relative wall thickness < 0.42.

[b] Concentric remodeling: LVMI ≤ 51 $g/m^{2.7}$ and relative wall thickness ≥ 0.42.

[c] LVH with eccentric remodeling: LVMI > 51 $g/m^{2.7}$ and relative wall thickness < 0.42.

[d] LVH with concentric remodeling: LVMI > 51 $g/m^{2.7}$ and relative wall thickness ≥ 0.42.

CHO = carbohydrate; LVH = left ventricular hypertrophy; LVMI = left ventricular mass index.
